# K^+^ Block Is the Mechanism of Functional Asymmetry in Bacterial Na_v_ Channels

**DOI:** 10.1371/journal.pcbi.1004482

**Published:** 2016-01-04

**Authors:** Van Ngo, Yibo Wang, Stephan Haas, Sergei Y. Noskov, Robert A. Farley

**Affiliations:** 1 Department of Physics and Astronomy, University of Southern California, Los Angeles, California, United States of America; 2 Department of Biological Sciences, Centre for Molecular Simulations, University of Calgary, Calgary, Alberta, Canada; 3 Department of Physiology and Biophysics, and Department of Biochemistry and Molecular Biology, Keck School of Medicine, University of Southern California, Los Angeles, California, United States of America; Weill Medical College of Cornell University, UNITED STATES

## Abstract

Crystal structures of several bacterial Na_v_ channels have been recently published and molecular dynamics simulations of ion permeation through these channels are consistent with many electrophysiological properties of eukaryotic channels. Bacterial Na_v_ channels have been characterized as functionally asymmetric, and the mechanism of this asymmetry has not been clearly understood. To address this question, we combined non-equilibrium simulation data with two-dimensional equilibrium unperturbed landscapes generated by umbrella sampling and Weighted Histogram Analysis Methods for multiple ions traversing the selectivity filter of bacterial Na_v_Ab channel. This approach provided new insight into the mechanism of selective ion permeation in bacterial Na_v_ channels. The non-equilibrium simulations indicate that two or three extracellular K^+^ ions can block the entrance to the selectivity filter of Na_v_Ab in the presence of applied forces in the inward direction, but not in the outward direction. The block state occurs in an unstable local minimum of the equilibrium unperturbed free-energy landscape of two K^+^ ions that can be ‘locked’ in place by modest applied forces. In contrast to K^+^, three Na^+^ ions move favorably through the selectivity filter together as a unit in a loose “knock-on” mechanism of permeation in both inward and outward directions, and there is no similar local minimum in the two-dimensional free-energy landscape of two Na^+^ ions for a block state. The useful work predicted by the non-equilibrium simulations that is required to break the K^+^ block is equivalent to large applied potentials experimentally measured for two bacterial Na_v_ channels to induce inward currents of K^+^ ions. These results illustrate how inclusion of non-equilibrium factors in the simulations can provide detailed information about mechanisms of ion selectivity that is missing from mechanisms derived from either crystal structures or equilibrium unperturbed free-energy landscapes.

## Author Summary

In this paper we show that bacterial voltage-gated sodium channels (Na_v_) conduct ions differently in the inward direction and in the outward direction because extracellular K^+^ blocks these channels. The mechanism of block by K^+^ is revealed in the simulations only when effects of applied forces in the inward direction are considered appropriately. The block of bacterial Na_v_ channels by K^+^ explains the experimental findings that inward K^+^ current in bacterial Na_v_ channels is very small under conditions where most mammalian Na_v_ channels conduct significant K^+^ current. The block by K^+^ ions can occur in the bacterial Na_v_ channels but is very unlikely to occur in mammalian channels due to differences in the amino acid sequences of the selectivity filters of the different channels.

## Introduction

Voltage-gated Na^+^- selective (Na_v_) and K^+^- selective (K_v_) ion channels provide the molecular pathways for most ion current flow across cell membranes during electrical activity in biological systems [1]. The sequential opening and closing of these channels in response to changes in the membrane potential leads to the initiation and propagation of action potentials in both neurons and muscle cells, and dysfunctions of the channels characterize many inherited disorders of the heart, brain, skeletal muscle and other organs [2]. Ion currents through both Na_v_ and K_v_ channels have been measured using electrophysiological techniques for many years, and the elucidation of molecular structures of ion channels over the past 15 years has provided structural and mechanistic foundations for understanding these currents. Molecular dynamics (MD) simulations have extensively characterized selective ion permeation in K^+^-selective channels primarily because of the availability of multiple K^+^-selective channel structures at atomic resolutions [3–6].

Although extensive electrophysiological measurements have been made for eukaryotic Na_v_ channels, there are currently no molecular structures available for eukaryotic Na_v_ channels. Published structures of Na^+^-selective channels have been obtained from bacterial Na_v_ channels [7–10]. The bacterial Na_v_ channels are composed of four polypeptide subunits, similar to K_v_ channels, whereas mammalian Na_v_ channels are composed of a single polypeptide. Molecular dynamics simulations of ion permeation through bacterial Na_v_ channels have predicted unperturbed-free-energy barrier differences for permeation of Na^+^ and K^+^ of 1–3 kcal/mol that are consistent with estimates for the P_Na_/P_K_ permeability ratio of 20–40 obtained from experimental values for reversal potentials obtained for eukaryotic Na_v_ channels under bi-ionic conditions[11–16]. Recently published electrophysiological data from bacterial Na_v_ channels, however, indicate that bacterial Na_v_ channels differ somewhat from eukaryotic Na_v_ channels. Finol-Urdaneta et al., for example, have observed that in the presence of 140 mM intracellular Na^+^ and 140 mM extracellular K^+^, the NaChBac channel is strongly outwardly rectifying for K^+^, with a reversal potential for K^+^ current obtained by extrapolation from the linear portion of the I-V plot approximately 40 mV less negative than the actual value of the reversal potential obtained from instantaneous I-V data [17]. These investigators also observed that the P_K_/P_Na_ permeability ratio differed by a factor of 10 with oppositely directed Na^+^/K^+^ gradients, which they characterized as a functional asymmetry of the channel. Similarly, Ulmschneider et al. were unable to detect any voltage-dependent inward K^+^ current through bacterial Na_v_Ms channels expressed in HEK 293 cells in the presence of 150 mM extracellular KCl, consistent with a large energy barrier to inward K^+^ flux through this channel [11]. These experimental results show that potassium ions are prevented from entering the selectivity filter from the extracellular side of the membrane, and that transmembrane voltages of 78–128 mV are needed to overcome the block of potassium ion current.

To elucidate the mechanism of block of the K^+^ current and to see if this mechanism has consequences for ion selectivity, we simulated the permeation of multiple sodium and potassium ions through the Na_v_ channel of *Arcobacter butzleri* (Na_v_Ab), and we calculated the work needed to move the ions through the channel. The results of these non-equilibrium simulations were combined with results from calculations of two-dimensional equilibrium potential of mean forces (PMF) generated by umbrella sampling and the Weighted Histogram Analysis Method (WHAM) [18, 19] to characterize the energetics of the biased movements over the equilibrium PMFs. The results explain the absence of measurable inward potassium currents through the bacterial Na_v_ channels at membrane potentials less negative than -70 to -100 mV [11, 12, 14, 17], and shed light into the effects of applied forces on ion interactions in bacterial Na_v_ channels. The mechanism of ion selectivity in bacterial Na_v_ channels that emerges from these results differs from those identified from either crystal structures or equilibrium free-energy landscapes, and is consistent with electrophysiological measurements of bacterial Na_v_ channels.

## Methods

**Fig 1:**
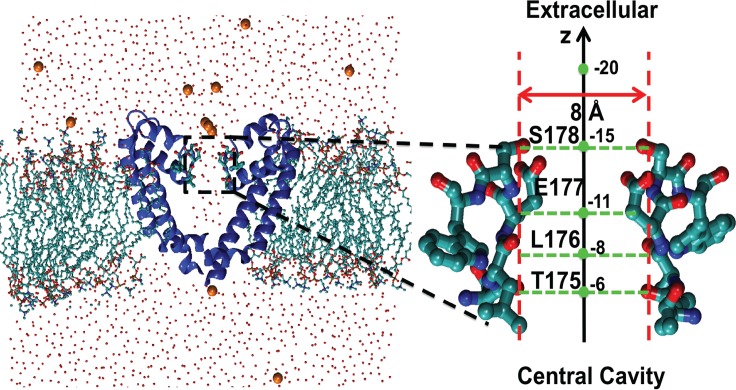
Simulation system of the sodium channel Na_v_Ab embedded in a lipid bilayer. For clarity, the voltage sensor domains are not shown; only two monomers, a few ions, and a thin layer of water are shown. The right panel depicts the selectivity filter lined by the amino acids TLESW. The green dots represent z-coordinates along the central axis, whose arrow indicates the inward direction. The distance of 8 Å between the oxygen atoms of opposite hydroxyl groups of S178 is greater than the smallest width of the selectivity filter, which is 4.6 Å.

The simulation system was constructed using VMD [20] and the simulations were run using NAMD [21], CHARMM27 [22, 23] for proteins with NBFIX [24] and CHARMM36 [25] for lipids. A crystal structure of Na_v_Ab (PDB: 3RVY) [9] was placed in the middle of a 40-ns pre-thermalized lipid bilayer containing 288 POPC molecules that was surrounded by two thin layers of water. Any lipid and water molecules within 1.4 Å of the protein were removed. Two extra water layers with a thickness of 24 Å were then added to the top and bottom of the membrane. 113 Na^+^ and 121 Cl^−^ions were added to establish electroneutrality (Fig 1). The total number of atoms in the simulation system is 186550. Systems containing KCl were constructed by changing Na^+^ to K^+^ after the system with NaCl was equilibrated.

The simulation system with NaCl was minimized for 5000 steps, and then the Gaussian velocity distribution at 300° K was used to start NVT for 10000 steps with a time step of 2 fs. During the initial equilibration of the system, the protein and membrane were constrained, and water molecules were excluded from the lipid bilayer. Next, NPT ensemble was used for a 200 ps equilibration run to further relax the protein-membrane system, with gradual removal of the constraints acting on the protein. All restraints on the protein were then removed and a harmonic potential with a spring constant of 10 kcal/mol/Å was applied to all atoms of the lipid bilayer. This strong constraint prevents the lipid membrane from moving during pulling simulations, while all atoms of Na_v_Ab are free to move, thus allowing us to focus on the movements of ions and all atoms of Na_v_Ab. The whole system was then thermalized for 1 ns. In these simulations, the time step was set to 1 fs. A Langevin thermostat was used to control temperature at *T* = 300° K with a damping constant of 1 ps^-1^ acting on atoms other than hydrogen, and Langevin piston dynamics were used to control pressure at *P* = 1 atm. The dimensions of the system after this equilibration step were 13.1×13.3×10.4 nm^3^.

After equilibration, step-wise pulling protocols [26–28] were used for non-equilibrium simulations of single-ion and three-ion configurations being pulled from the extracellular side of the membrane to the central cavity of the channel. The choice of the three-ion system was based on recent papers that investigated permeation mechanisms in depth. Microsecond simulations of ion permeation in Na_v_Ab at zero voltage by Chakrabarti et al. [15] and Boiteux et al. [16] indicate that the conduction state involves three Na^+^ ions in the selectivity filter; thus we emphasized the movements of three ions in these simulations (see Discussion). Either a single ion or three ions were placed in the extracellular solution near the entrance of the selectivity filter at *z* = -20Å (see Fig 1). The selectivity filter extends from *z* = –15 Å at S178 to *z* = –5 Å at T175. Independent harmonic potentials *U*(*z*_*i*_,*λ*_*i*_) = 0.5*k*(*x*–*x*_0_)^2^ + 0.5*k*(*y*–*y*_0_)^2^ + 0.5*k*(*z*– *λ*_*i*_)^2^ were applied to the ions, where (*x*,*y*,*z*) are the coordinates of an ion, *x*_0_ = *y*_0_ = 0 Å is the position of the z-axis of the symmetric system, *k* = 0.6 kcal/mol/Å^2^ ~ 1 kcal/mol/Å^2^, λ is the center of the harmonic pulling potential, *i* is the *i*-th pulling step from 0 to 18, and *λ*_*i*_ is increased by 1.0 Å from –20Å to –2Å. For a deviation of 2 Å from the z-axis, the harmonic potentials on the xy-directions introduce a weak bias of only 1.2 kcal/mol (~2k_B_T), which is within a thermal fluctuation. These constraints are used to control lateral displacement of an ion at the entrance to the selectivity filter and to enhance essential ionic dynamics along the z-axis. These potentials are used to mimic a possible gradient of ionic concentration on xy-plane so that ions “prefer” to move into the pore of Na_v_Ab instead of being trapped by dipoles of lipid head-groups. If the energetic barrier for two ions in the xy-directions is much higher than 1.2 kcal/mol, the ions are likely to line up along the z-axis (see Discussion). Interestingly, our simulations show that even though the XY potentials are used to enhance a single-file pattern, two and three potassium ions can still orient themselves in almost the same xy-plane. The increment of 1.0 Å for *λ* has been shown to provide reliable estimates of useful work [27]. Following each increment of *λ*_*i*_, a relaxation period was introduced in which the coordinates of the atoms of the system were unconstrained (see below). The soft harmonic pulling potential is useful to characterize the dynamically mutual responses between ions and the selectivity filter [26, 28]. To remove any bad-energy contacts between the pulled ions and water molecules, energy minimization was run for 1000 steps at *λ*_0_ = –20 Å, and NPT coupling was used for the rest of the simulations. No minimization step was done for the pulling steps *i* > 0. Two sets of sequential pulling simulations were performed by using the configuration at the end of each relaxation time corresponding to *λ*_*i*_ as the initial configuration for *λ*_i+1_. In one set of simulations, *λ* was instantaneously increased every 0.5 ns to mimic fast movement of ions throughout the selectivity filter. The final configuration at *λ*_0_ = –20 Å was then used as the initial configuration to perform another set of sequential pulling simulations in which the relaxation period was increased to 2.5 ns to capture slower dynamics of ions. During the total relaxation time of 3 ns for each value of *λ*, the positions of ions and external forces of ions were recorded every 50 fs to compute work distribution functions. The data generated from these pulling simulations are generally non-equilibrium compared with the dwelling time of 0.1 to few microseconds of K^+^ ions found in Refs. [15] and [16].

Jarzynski’s Equality was used to exponentially weight trajectories sampled in the work distribution functions to estimate a perturbed Helmholtz free-energy change. In this way, the free-energy change indicates useful work, which takes into account entropic effects, to move ions along a pathway. It is directly related to the work done by either ion concentration gradients or applied voltages to drive ions through the channel. In the step-wise pulling protocols, relaxation times are used to sample possible stable binding sites, to allow certain slow transitions between stable binding sites to occur, and to dissipate external energies into the system. Depending on the system, the convergence of perturbed Helmholtz free-energy changes can occur fairly quickly. For a small system such as deca-alanine, a convergent free-energy change can be achieved with relaxation times as small as 0.4 ns [27]. For a system such as KcsA (PDB 1K4C) [26] and an 8-base nucleic acid G-quadruplex [28], relaxation times from 3 ns to 5 ns have been found to accurately distinguish the free-energy change of K^+^ from that of Na^+^ and to identify the differences in their selectivity mechanisms. The variance associated with the free-energy changes computed from this method can be estimated from [σ^2^_W_/Q+σ^4^_W_(k_B_T)^-2^/2(Q-1)]^1/2^ ≈ 1.0 kcal/mol, where σ_W_ is a standard deviation of a work distribution function and *Q* is a number of data points in the work distribution. As shown below, a relaxation time of 3 ns can identify significant differences in the perturbed Helmholtz free-energy changes of three ions in Na_v_Ab, can provide mechanistic information about ion selectivity in Na_v_ channels, and can explain the essential impermeability of Na_v_Ab and other bacterial Na_v_ channels to inward movement of K^+^ ions. The simulation procedures used to obtain potential of mean forces (equilibrium unperturbed free-energy landscapes) via umbrella sampling and Weighted Histogram Analysis methods are given in [17].

## Results

### Single ion simulations

**Fig 2:**
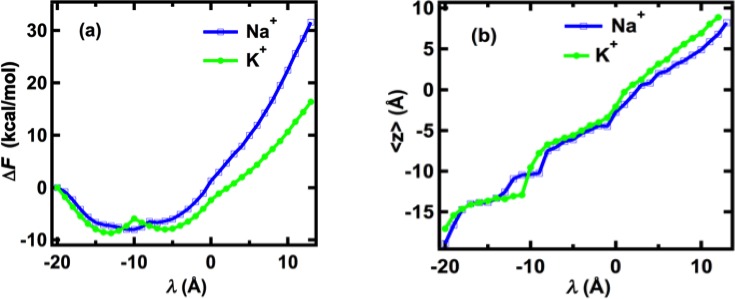
(a) Perturbed Helmholtz free-energy changes (useful work) of single K^+^ and Na^+^ ions pulled through the Na_v_Ab channel with relaxation times equal to 0.5 ns for each pulling step. The value of *λ* indicates the center of the harmonic pulling potential (see Fig 1). (b) Averaged z-coordinate of single K^+^ and Na^+^ ions. The z-coordinates more negative than –15 Å are on the extracellular side of the membrane, and z-coordinates greater than 10 Å are on the intracellular of the membrane.

Other investigators have reported results from simulations of single ion permeation through Na_v_ channels [12, 14, 17]. The conclusions reached by computing the potential of mean forces are that single ions do not permeate rapidly through Na_v_ channels, and that the presence of at least two hydrated Na^+^ ions reduces energy barriers and binding affinities and facilitates permeation. Here we briefly summarize the results of single ion step-wise pulling simulations primarily to compare the results of the single-ion configuration and the three-ion pulling configuration later. Fast pulling simulations were performed on single Na^+^ or K^+^ configurations from the extracellular side of the membrane at *z*_ex_ = –20 Å to the intracellular side at *z*_in_ = 10 Å using a relaxation time of 0.5 ns for each pulling step. With an increment (Δ*λ*) of the center of the harmonic pulling potential (*λ*) of 1.0 Å along the axis of the pore during each pulling step, this corresponds to an average speed of about 2.0 Å/ns for ions to move through the channel. The free-energy changes generated from these pulling simulations shown in Fig 2A indicate that the single Na^+^ and K^+^ ions experience free-energy minima and barriers at different positions along the axis of the channel. This result is consistent with results obtained from calculation of the potential of mean forces [14].

The single K^+^ ion enters the selectivity filter at a local minimum at λ = –13 Å and <*z*> = –13.4 Å. Further into the selectivity filter another local minimum is located at λ = –5 Å, <*z*> = –5.6 Å, and between those minima the K^+^ ion experiences a free-energy barrier of 2.8 kcal/mol with respect to the first minimum. The single Na^+^ ion moves to the minimum at <*z*> = –10.4 Å (λ = –10 Å) without an energy cost, but requires larger work than the single K^+^ ion to translocate further into the central cavity. Note that the position <*z*> = –10.4 Å is the location of the carboxyl groups of E177. Although the fast pulling simulations of the single ions cannot be used to demonstrate the selectivity of Na^+^ over K^+^ due to the nature of multiple-ion permeation in Na_v_Ab, they do demonstrate that E177 attracts Na^+^ and that K^+^ experiences a free-energy barrier when moving deep into the selectivity filter.

### Three-ion pulling configurations

**Fig 3:**
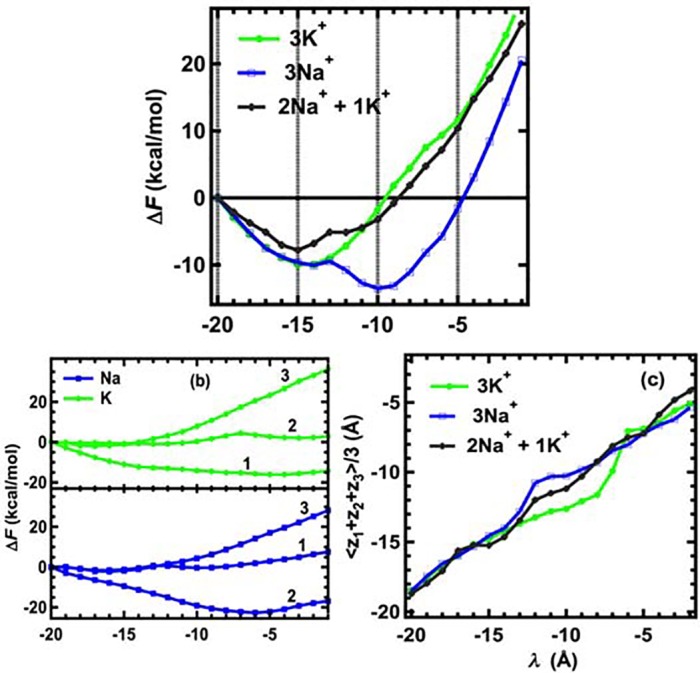
(a) Perturbed Helmholtz free-energy change (useful work) of three-ion pulling configurations. The value of *λ* indicates the center of the harmonic pulling potential (see Fig 1). (b) Perturbed Helmholtz free-energy change (useful work) of individual ions in the three-ion pulling simulations. The numbers indicate the ordered ions in the three-ion configurations. (c) Averaged z-coordinate of three ions.

The single-ion simulations indicate that fast pulling or a 0.5 ns relaxation time does not differentiate between the movements of sodium and potassium ions. Because microsecond simulations by Chakrabarti et al. [15] and Boiteux et al. [16] suggested that the conduction state of the Na_v_Ab channel involves three Na^+^ ions, we performed three-ion pulling simulations with averaged pulling speed of 2.0Å/ns (0.5 ns relaxation time) to determine whether Na^+^ permeation can be distinguished from K^+^ permeation under these conditions. The results (not shown) indicated that these fast pulling simulations were also unable to show clear differences in the movements of two ionic species leading to ion selectivity. Consequently, a relaxation time of 3 ns between pulling steps in the three-ion pulling simulations was used to obtain more reliable statistics. This relaxation time corresponds to an average pulling speed of 1/3 Å per ns. As shown in Fig 3A, a free-energy minimum is observed for both three Na^+^ ions and three K^+^ ions at λ ≈ –15 Å, at the entrance to the selectivity filter. At this value of *λ*, the averaged position of the three ions is <*z*_1_+ *z*_2_+ *z*_3_>/3 ≅ –15 Å (Fig 3C). From this position, the K^+^ ions encounter an energy barrier that hinders further entry into the selectivity filter such as was observed in the case of a single ion (Fig 2A). The three Na^+^ ions, however, move into a more stable free-energy minimum, –13.5 kcal/mol, at λ = –10 Å and <*z*_1_+ *z*_2_+ *z*_3_>/3 ≅ –10 Å. The pulling force on each ion is proportional to the distance between the center of the harmonic pulling potential λ and the position of the ion z, (*U*(*z*_*i*_,*λ*_*i*_) = 0.5*k*(*z*– *λ*_*i*_)^2^). Increasing the pulling force by increasing *λ* from -15Å to –12Å causes the three Na^+^ ions to move further into the selectivity filter from z = –15 Å to z = –10Å, whereas the K^+^ ions do not advance to z = –10Å until *λ* ≈ –7Å. At λ = –5Å, the free-energy change of 3K^+^ is significantly higher than that of 3Na^+^ (Fig 3A). From λ = –20 Å to *λ* = –5 Å, the work done to bring 3K^+^ from an averaged position <*z*_1_+ *z*_2_+ *z*_3_>/3 = –19 Å (extracellular) to –7 Å (~ central cavity) is about 11.6 ± 1 kcal/mol, whereas it is –1.5 ± 1 kcal/mol for 3Na^+^. The difference of about 13 kcal/mol between the two free-energy profiles at λ = –5 Å is the difference in the amount of work required to pull 3Na^+^ ions and 3K^+^ ions through the selectivity filter of Na_v_Ab. These values indicate that while it requires a significant amount of work to move three K^+^ ions through the selectivity filter of Na_v_Ab, the movement of three Na^+^ ions from the extracellular side of the membrane to a position very close to the central cavity of the Na_v_Ab channel is thermodynamically favored by as much as 1.5 kcal/mol. This result explains the observed impermeability of Na_v_Ab for inward K^+^ current (see below) [11, 17]. The free-energy changes shown in Fig 3B further depict how each individual ion interacts with other ions and with the environment during permeation through Na_v_Ab. The last ions (K_3_ and Na_3;_ denoted by the number 3 in Fig 3B) experience the highest free-energy barriers due to the presence of the other ions. The net useful work for the first two Na^+^ or K^+^ ions is negative, confirming the results obtained from calculations of PMF that multiple ions interact more stably with the channel than single ions, although the order of ion binding influences the values of the useful work. For K^+^, the free-energy change of the second ion K_2_ is more flat than that of Na_1_, indicating the ease of translocating this K^+^ to a stable binding site outside the selectivity filter, whereas for Na^+^, the second ion (Na_2_) falls into a deep energy trough in the presence of Na_1_. This energy trough is not present for a single Na^+^ ion (Fig 2). These observations are also consistent with the conclusions of others that the selectivity filter responds more effectively to multiple ions than a single ion [12, 17]. The second sodium ion Na_2_ experiences the largest negative ΔF change in comparison with the other ions.

**Fig 4:**
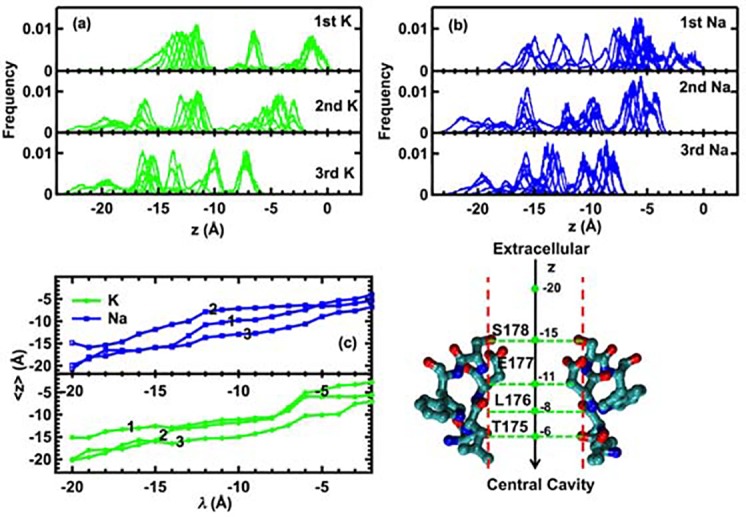
(a-b) Histogram of z-coordinate positions of all ions during the three-ion inward pulling protocol. (c) Averaged z-coordinate of each ion in the three-ion configurations during the pulling simulations. The numbers indicate the same ordered numbers in (a-b). The Na_v_Ab selectivity filter is shown on the right side of the Fig for reference.

Fig 4 shows frequency histograms for the positions of the ions in the selectivity filter of Na_v_Ab during all pulling steps. Peaks in the histograms correspond to stable positions for the ions in the selectivity filter. The histograms of the three individual K^+^ ions in Fig 4A resemble a series of single Gaussian distributions around the average positions for each ion, and the peaks are more widely separated than those of the three individual Na^+^ ions shown in Fig 4B. The histograms corresponding to the Na^+^ ion distributions appear as clusters of multiple Gaussian distributions with considerable overlap between Gaussians. The distribution of separated peaks of the K^+^ ions suggests that K^+^ ions in the Na_v_Ab selectivity filter may prefer individual stable and separated binding sites like those found in the selectivity filters of potassium channels. The overlaps in the histograms for the individual Na^+^ ions indicate that Na^+^ ions can move frequently along the z-axis and in the x,y-plane of the selectivity filter between different binding sites containing oxygen atoms from carboxylate (E1777), carbonyl (L176) and hydroxyl (S178) groups.

Fig 4C shows how 3Na^+^ ions and 3K^+^ ions move together through the Na_v_Ab selectivity filter. Initially at λ = –20 Å, 3Na^+^ ions approach the selectivity entrance in the same configuration as the 3K^+^ ions, with one ion near the hydroxyl groups of S178 at *z* = –15 Å and two ions located at the same coordinate *z* = –20 Å. This configuration mimics an ion gradient that helps to drive both Na^+^ and K^+^ ions further into the selectivity filter in the same manner until λ = –15 Å. After this point, the two ionic species behave differently, particularly at λ = –12 Å. K_1_ and K_2_ have the same z-coordinate for λ = –14 Å to –4 Å, while K_3_ lags slightly behind them. In contrast, 3Na^+^ ions are arranged about 3 Å apart, projected onto the z-axis, and move together as a unit through the selectivity filter. This coordinated movement of 3Na^+^ ions mimics a “knock-on” mechanism of permeation, and is evident around E177. A “knock-on” mechanism for permeation is also observed in other simulations of sodium channels and potassium channels. In the next sections we show how the carboxylate groups of two adjacent E177 residues deliver 3Na^+^ ions through the selectivity filter.

### Potassium blocks the selectivity filter of Na_v_Ab

**Fig 5:**
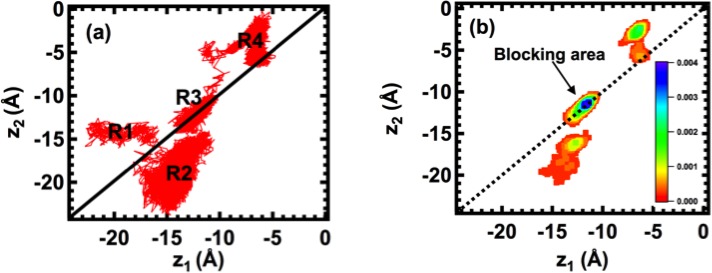
(a) z-coordinate of K_1_ (horizontal axis; z_1_) versus z-coordinate of K_2_ (vertical axis; z_2_) during the pulling simulations. (b) Frequency of data points in (a).

Fig 5A shows how two potassium ions, K_1_ and K_2_ in Fig 4C, move through Na_v_Ab with respect to one another, and Fig 5B shows the frequencies of the data points in Fig 5A.

K_2_ first approaches S178 at *z*_2_ ≅ –14 Å and lingers there, while K_1_ moves around in the mouth of the selectivity filter between -14 Å ≤ z ≤ -23 Å, as shown in region R1. Region R3 is close to the ring of negative charges in the selectivity filter formed by the carboxylate groups of E177, but the K^+^ ions do not enter region R3 directly from region R1. Instead, K_1_ displaces K_2_ and moves further into the selectivity filter, approaching E177, as K_2_ moves back between -14 Å ≤ z ≤ -23 Å (region R2). Both K_1_ and K_2_ then move to the same z-coordinate in region R3, which is identified as a stable site with the highest probability for the ions, and is identified in Fig 5B as a “blocking area”. When applied forces increase (Fig 4C), K_1_ remains in the blocking area until K_2_ has moved deeper into the selectivity filter, and then both of the ions escape the selectivity filter at *z* ≅ –5 Å.

**Fig 6:**
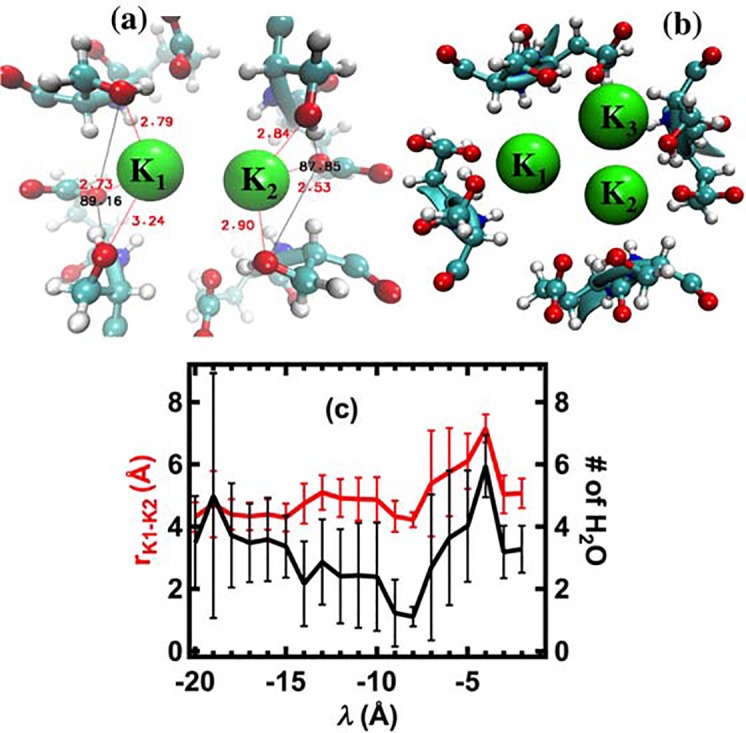
Snapshots of two (a) and three (b) K^+^ ions binding to oxygen atoms of carboxylate and two hydroxyl groups of E177 and S178 at λ = –10 and –9 Å, respectively. The views are from the extracellular side of the membrane. The red numbers are distances (Å) between the side chain oxygen atoms of E177 and S178 and the ions. The black numbers are angles of O_S_–O_E_–O_S_. (c) Averaged distance and number of water molecules between K_1_ and K_2_. The data of 2.5 ns in each pulling step are collected for the averages. The number of water molecules is counted in the overlap between the two spheres, which have instantaneous radius r_K1-K2_ and are centered at the positions of the ions.

As shown in Fig 5B, the blocking area encompasses about 3–4 Å along either the z_1_- or z_2_-axis, indicating that the selectivity filter can respond to movements of the K^+^ ions over a distance of 3–4 Å. This distance is about one third of the length of the selectivity filter. Fig 5B also shows that the blocking area is asymmetric with respect to the K^+^ binding sites. The lower area (region R2) is closer to the blocking position than the upper one (region R4), suggesting that the K^+^ ions are more prone to fall into the blocking area when moving from the extracellular surface of the membrane to the cytoplasmic surface than moving in the reverse direction. Fig 6A shows a snapshot of the block of the selectivity filter by two K^+^ ions coordinated by carboxylate oxygen atoms of E177 and neighboring hydroxyl groups of S178. When viewed from the extracellular side of the membrane, oxygen atoms of hydroxyl and carboxylate groups of neighboring S178 and E177 residues coordinate the K^+^ ions by a network of hydrogen bonds between them to trap the ions. When viewed from the intracellular side of the membrane, however, the carbonyl oxygen atoms of L176 and the hydroxyl groups of T175 form a backbone arrangement similar to that of KcsA, although the two channels do not coordinate the K^+^ ions in the same way. This asymmetry in the binding of the K^+^ ions may explain why the Na_v_Ab channel is much less permeable to the inward flux of K^+^ ions than to outward K^+^ current. This asymmetry is also consistent with the observations of Ulmschneider et al. who observed in unbiased molecular dynamics simulations that extracellular Na^+^ ions move through the selectivity filter, although the number of permeation events was small, whereas extracellular K^+^ ions appear to halt at a position near E177, with a large energy barrier preventing further inward translocation [11].

To describe how two K^+^ ions effectively block the selectivity filter, the averaged distance between the ions and the number of shared water molecules between the ions were examined for all pulling steps (Fig 6C). Before the K^+^ ions enter the blocking area, the averaged distance between them is approximately 4.5 Å, which is approximately equal to the smallest diameter of the selectivity filter (4.6Å). The K^+^ ions share about four water molecules that screen the repulsive interaction between them. When the ions enter the blocking area (–15 Å ≤ λ ≤ –10 Å), the distance between them increases by approximately 0.5 Å and the number of shared-water molecules decreases by two. The averaged distance of 5.0 Å and partial dehydration of the K^+^ ions leads to effective blocking of the channel because the ions repel one another with a force of approximately 14 kcal/mol/Å (~ 1.0 nN) perpendicular to the lateral walls of the selectivity filter. As a result, the third K^+^ ion (K_3_) is less effective in knocking K_1_ and K_2_ further into the selectivity filter, and instead falls into another binding site formed by oxygen atoms of neighboring E177 and S178 amino acids (Fig 6B). To break the block of the channel due to the presence of the three K^+^ ions, energy must be provided to drive the ions through the selectivity filter. The energy needed to break the block under the conditions of the simulations is about 5–10 kcal/mol, shown in Fig 3A as the ΔF between λ = –8 Å to –6 Å. It can be seen in Fig 3C that between these values of λ, the 3K^+^ ions break from the position of the block in the middle of the blocking area at about <*z*> = -12Å and move out of the blocking area to a position at about z = -8Å.

In electrophysiological experiments, energy to break the K^+^ block comes from the membrane potential. For membrane potentials less negative than about –100 mV = –6.9 kcal/mol/3|e| (about the breaking energy), there is insufficient energy available to move three K^+^ ions through the selectivity filter in the inward direction. Hence, there will be no inward current of K^+^ ions. The membrane potential that is sufficient to both break the block by extracellular K^+^ and to drive the inward flux of K^+^ corresponds to an applied potential (~ -100 mV) of the channel for K current in the instantaneous I-V plot [11, 17].

### Permeation of Na^+^ ions through Na_v_Ab

**Fig 7:**
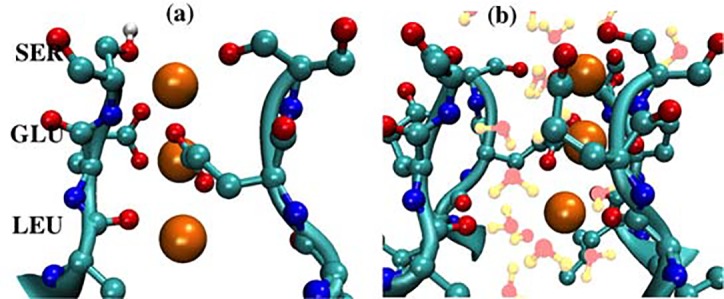
(a) Snapshot of three Na^+^ ions aligned along one lateral corner of adjacent SEL amino acids at λ = –12Å. (b) Snapshot of all atoms in (a) showing water molecules that fill the rest of the selectivity filter.

In contrast to the blocking state of 3K^+^ ions, 3Na^+^ ions form a single file parallel to the axis of the channel pore to enable a loose “knock-on” mechanism of permeation. The simulations show that the Na^+^ ions can bind to oxygen atoms of carboxylate, hydroxyl and carbonyl groups of adjacent S178, E177 and L176 residues on one subunit. We observed that the carboxylate groups of two adjacent E177 amino acids can either “hold” a single Na^+^ ion or “pass” it between them. Such flexible motion of the carboxylate groups creates and stabilizes a single-file permeation of 3Na^+^ ions in our simulations, which occurs at the position of the free-energy minimum (λ = –10 Å), i.e., this single file is an optimal translocation configuration for Na^+^ ions. As seen in Fig 4C, 3Na^+^ ions then move together in the specific order. As has also been reported by others [11, 12, 14], the Na^+^ ions align off the z-axis of the pore, in this case along one lateral corner of the selectivity filter, where the oxygen atoms of adjacent SEL amino acids are located (Fig 7).

The opposite corner of the selectivity filter, having the other SEL amino acids, appears to be coordinated by water molecules. This opposite lateral corner might play a back-up role to trap any K^+^ ions moving with Na^+^ ions because it has a similar hydrogen bond network generated by S178 and E177 amino acids. That the free-energy change for one K^+^ and two Na^+^ ions (Fig 3) is higher than that of 3Na^+^ ions is consistent with this possibility. A back-up role is also consistent with experimental findings that K^+^ ions can compete with Na^+^ ions to reduce ionic current [29]. Note that the movement of 3Na^+^ ions along one lateral corner does not rule out the possibility that they can switch to the other three equivalent lateral corners, which are also composed of adjacent LES amino acids.

### 2D potentials of mean forces

**Fig 8:**
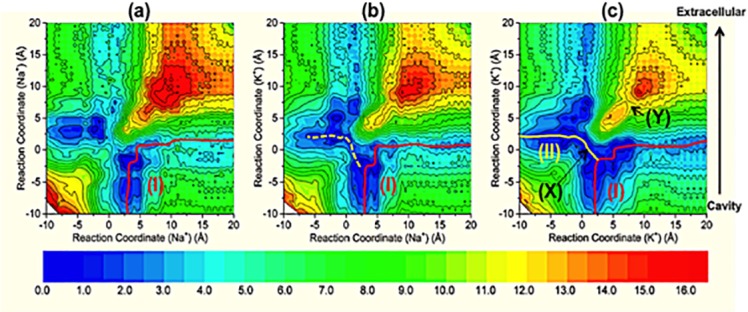
Two-dimensional PMF (unperturbed equilibrium free-energy landscape) of Na-Na (a), Na-K (b) and K-K (c) along the selectivity filter of Na_v_Ab. The zero coordinate (*z’*) is at the center of mass of backbone atoms of ELT, around the positions of L176 carbonyl oxygen atoms (*z’* = -8 Å in Fig 4). The solid red line (I) describes a “knock-on” pathway of permeation for the ions pairs. The dashed yellow line (Fig 8b) indicates a path energetically less favorable than the solid yellow line (II; Fig 8c), which is energetically equivalent to (I). (X) denotes the global minimum, which has the two potassium ions at the same z-coordinate. (Y) denotes the unstable local free-energy minimum seen along the diagonal (*z’* ≈ -14 Å in Fig 4).

Two-dimensional plots of PMF for Na-Na, Na-K, and K-K ion pairs along the selectivity filter of NavAb are shown in Fig 8.

The PMF of Na^+^-Na^+^ pair in Fig 8A identifies a favorable path for the two Na^+^ ions through the selectivity filter from the extracellular surface of the membrane to the cytoplasm (solid red line; pathway I). This path describes a “knock-on” mechanism of permeation in which the two Na^+^ ions are aligned along the Z-axis of the channel at different z-coordinates. A “pass-by” configuration [16] of two sodium ions in which the ions occupy the same z-coordinate is about 3–4 kcal/mol less favorable than the “knock-on” configuration. Pathway (I) shows that Na^+^ ions move in the inward direction without noticeable extra energy cost. This result is consistent with the free-energy differences calculated in the step-wise pulling simulations. The replacement of one Na^+^ ion with a K^+^ ion to form a Na^+^-K^+^ pair of ions (Fig 8B) reduces the barrier to the “pass-by” configuration for the Na^+^-K^+^ pair by 1–2 kcal/mol; thus the “pass-by” configuration will occur more frequently than in the case of two sodium ions. Permeation pathway (I) is still favored energetically for a pair of K^+^ and Na^+^ ions but the pathway shown in Fig 8B as a dashed yellow line may occur more frequently for the Na^+^-K^+^ ion pair than for the Na^+^-Na^+^ ion pair. The PMF profile of the K^+^-K^+^ pair in Fig 8C shows a free energy minimum where the two K^+^ ions are located at the same z-coordinate in the “pass-by” configuration (X; Fig 8C). This is a global free-energy minimum. Two potassium ions can enter into this free-energy minimum and follow either pathway (I) or pathway (II) to enter the central cavity of the channel. Since there is no energy barrier found at the crossing of the two pathways, the two pathways are energetically equivalent.

The two K^+^ ions at (X) can easily move from position (X) since the barriers to migration are comparable to energy stored in the thermal bath. One of two K^+^ ions can enter the cavity either through the pathway (I) or (II) without energy cost, and then another permeation cycle is initiated when another K^+^ ion moves to replace it at (X). As described above, probability of a K^+^ ion utilizing either pathway (I) or pathway (II) is approximately equal. The results of the PMF calculations, therefore, predict that in the presence of even a small inwardly directed [K^+^] gradient, a net inward K^+^ current would occur when the Na_v_Ab channel is fully open and activated. As described above, however, experiments show that a large inside-negative membrane potential (~ -100 mV) is required to induce inward currents of K^+^ ions in the fully open-activated state of Na_v_Ab channels [17], and the predictions derived from the equilibrium PMF calculations, therefore, are not consistent with the experimental observations. A model is presented in the Discussion that includes both the properties of the equilibrium free-energy landscapes and the results of non-equilibrium pulling simulations to describe the mechanism of potassium inward currents occurring at an applied potentials around -100 mV.

In Fig 8C, (X) denotes a stable global free-energy minimum at L176 near the entrance to the central cavity of the channel where two K^+^ ions can occupy a “pass-by” configuration at the same z-coordinate. In the 2D PMF, the region for two K^+^ ions at this position encompasses a distance of about 3 Å along the z-axis, which is similar to the block area identified in Fig 5B. Two K^+^ ions can also adopt a “pass-by” configuration at reaction coordinate (Y) in Fig 8C; however, (Y) denotes an unstable local free-energy minimum where the hydroxyl groups of S178 attract K^+^ ions.

### Functional asymmetry

**Fig 9:**
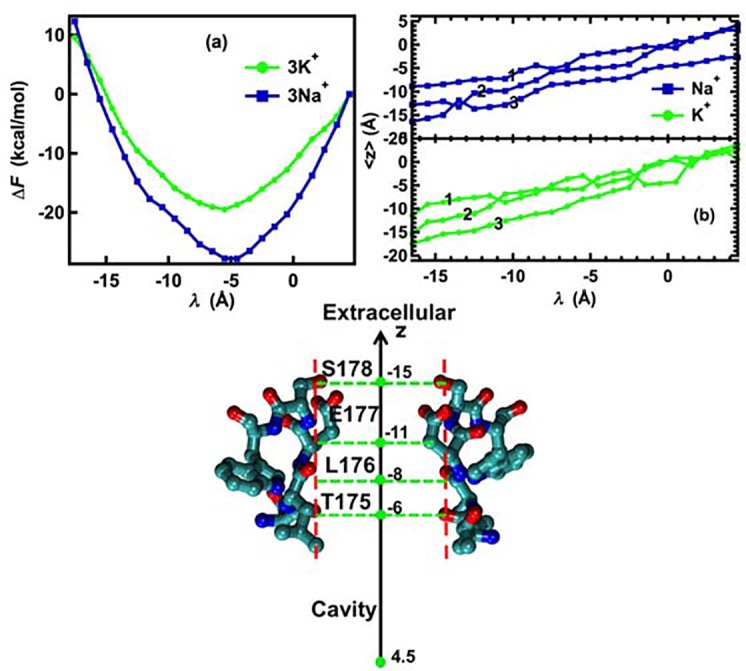
(a) Perturbed Helmholtz free-energy change (useful work) of three potassium and sodium ions pulled in the outward direction. (b) Average position of each individual ion in the outward pulling simulations. The ions move from the right to left of the horizontal axis. (c) The orientation and structure of the selectivity filter of Na_v_Ab is shown at the right side of the Fig.

In order to examine if the same block effect would occur for the ions moving in the outward direction, i.e., from the water cavity to the extracellular side, we started with the final configuration from the inward pulling simulations, and then sequentially pulled the ions from 4.5 Å to –17.5 Å using *τ* = 3 ns for each pulling step. Fig 9A shows the useful work required to move three of each ionic species through the selectivity filter from <*z*_1_ + *z*_2_+ *z*_3_>/3 ≈ 1.5 Å to –17 Å. The lower Δ*F* minimum for the sodium ions indicates that when approaching from the central cavity, the sodium ions bind more strongly to the selectivity filter than the potassium ions by 10 kcal/mol. This result and the result shown in Fig 3A suggest that moving in either the inward or the outward direction through Na_v_Ab, three sodium ions bind to the selectivity filter more strongly than three potassium ions. The potassium ions behave differently, however, when they enter the selectivity filter from the cavity compared to entry from the extracellular solution, i.e., no blocking effect is observed. Fig 9B shows that sodium ions continue to favor a “loose” knock-on mechanism in the selectivity filter (–15Å ≤ *z* ≤ –5Å) when approaching from the central cavity, although two sodium ions approach T175 (*z* ~ –5 Å) at the same time. Two potassium ions approach T175 in a manner similar to that observed for the two sodium ions, but one potassium ion is knocked into the selectivity before the other two, and thus is not able to form a block against the other ions. At *λ* = –5.5 Å, where the minima of the two ionic species are located, both sodium and potassium ions are in similar single-file configurations with (*z*_K1_, *z*_K2_, *z*_K3_) = (–3.0, –5.8, –7.9) and (*z*_Na1_, *z*_Na2_, *z*_Na3_) = (–2.4, –5.0, –7.9). Two potassium ions can also bind at almost the same z-coordinate near L176 (*z* ~ –8 Å), in agreement with the global free-energy minimum for two potassium ions near L176 (see Fig 8C). At the blocking area identified in the inward pulling simulations (–15Å ≤ *z* ≤ –10Å), however, the three potassium ions also prefer a single-file configuration, indicating that in bacterial Na_v_ channels, the selectivity filter can only block the potassium ions moving in the inward direction. Movements of potassium ions in the outward direction proceed relatively unhindered. Between *λ* = –10 and –16.5 Å, one potassium ion or one sodium ion is poised to exit the selectivity filter at *z* ≤ –15 Å. Particularly, at *λ* = -15.5Å, the total useful work to drive one of three Na^+^ ions out of the selectivity filter is almost zero. This suggests that if there is a continuous supply of ions to replace the exiting sodium ion, steady state sodium current requires no external energy. The outward movement of the three potassium ions, however, requires about 3 kcal/mol more than for sodium ions at the same value of *λ* = -15.5Å. The first potassium ion is poised to exit the selectivity at *λ* = –14.5 Å (<z_3_> ≈ –15 Å), however, where the useful work is less than zero. Thus, if there is a continuous flow of potassium ions in the outward direction, each group of three potassium ions would also encounter no free-energy barrier when translocating through the selectivity filter. At more negative values of *λ* and with no incoming ion to replace for the exiting ion, the absolute values of the useful work become as large as 10 kcal/mol for both ions. The work required to move the three sodium ions through the selectivity filter is also larger than that for the potassium ions under these conditions. This difference is probably due to the higher binding affinity of the selectivity filter for multiple Na^+^ than for K^+^ ions (Fig 9A). The mechanism described by these simulations is consistent with steady state outward currents for either Na^+^ or K^+^, as have been observed experimentally. Thus, we only observe a block of inward potassium current, but in the outward direction all three potassium ions can move through the selectivity filter much more easily than in the inward direction.

## Discussion

Despite differences in structure between mammalian and bacterial Na_v_ channels, calculations of PMF from equilibrium molecular dynamics simulations of ion permeation through bacterial Na_v_ channels have reproduced several experimental properties of mammalian Na_v_ channels such as the P_Na_/P_K_ permeability ratio. Although experimental measurements of reversal potentials for eukaryotic Na_v_ channels under bi-ionic conditions usually yield a P_Na_/P_K_ permeability ratio of 20–40 [1], recent measurements by Finol-Urdaneta et al. of instantaneous ionic currents and reversal potentials of potassium ions for the bacterial NaChBac channel identified a functional asymmetry that is not observed for most mammalian Na_v_ channels [17]. The amino acid sequence of the selectivity filter of the NaChBac channel is identical to the Na_v_Ab channel used in the simulations discussed in this report.

In the experiments of Finol-Urdaneta et al., no inward K^+^ currents were observed at membrane potentials more positive than about -100 mV for NaChBac when [Na^+^]_inside_ = 140 mM and [K^+^]_outside_ = 142.5 mM, whereas in symmetric high Na^+^ solutions, the I-V plot crossed the voltage axis at the origin. A P_Na_/P_K_ ratio of 5 was calculated from these measurements. A reversal potential close to +50 mV was measured when [K^+^]_inside_ = 140 mM and [Na^+^]_outside_ = 142.5 mM, and when measurements were made in symmetric high K^+^ solutions, the I-V plot also crossed the voltage axis close to the origin. The P_Na_/P_K_ ratio calculated from these data was 50. A kinetic asymmetry in the rate of displacement of tightly bound extracellular K^+^ by either internal Na^+^ or internal K^+^ was also observed for the NaChBac channel (see below).

Ulmschneider et al. [11] simulated the permeation of Na^+^ and K^+^ ions using the crystal structure of the bacterial Na_v_MS channel, and consistent with the results of Finol-Urdaneta et al., did not observe inward currents of K^+^ when [K^+^]_outside_ = 150 mM, under conditions where Na^+^ block could be ruled out. The Na_v_MS channel crystal structure differs from the crystal structure of the Na_v_Ab channel in that the Na_v_MS channel structure represents the open conformation of the Na_v_MS pore [7] whereas the cytoplasmic gate in the Na_v_Ab channel structure is closed [9]. Nevertheless, the structure of the selectivity filter in the two channels is very similar and the activation gate appears to be uncoupled from the selectivity filter [7].

Finol-Urdaneta et al. suggested that functional asymmetry might reflect a common property of all P-loop channels that possess a non-selective entrance to the internal cavity and a selectivity filter that is located closer to the extracellular mouth [17]. Simulations of ion permeation in both Na_v_Ab and Na_v_MS by Stock et al. [30] and Ke et al. [31] also indicate that the mechanisms of Na^+^ permeation in the inward direction and in the outward direction may differ.

The simulations described in this report indicate that the inability of extracellular potassium to move through the Na_v_Ab channel is due to blockage of the channel by two or three K^+^ ions that occurs in the presence of external forces such as would occur with small voltages or concentration differences across the membrane. Block of the bacterial Na_v_ channels by extracellular K^+^ is a good model for the experimental observations that no inward K^+^ current was observed through the bacterial Na_v_MS channel expressed in HEK 293 cells even when K^+^ was the only extracellular monovalent cation, or through the NaChBac channel expressed in mammalian tSA 201 cells in the presence of intracellular Na^+^, except at large negative membrane potentials [11, 17]. Interestingly, Finol-Urdaneta et al. observed that inward whole-cell Na^+^ currents through NaChBac were reversed when extracellular Na^+^ was replaced with extracellular K^+^, and were restored when the extracellular K^+^ was replaced by extracellular Na^+^ [17]. The time course of current changes measured during wash-in and wash-out protocols to substitute extracellular Na^+^ and K^+^ are consistent with high affinity binding of extracellular K^+^ in the channel where K^+^ is only slowly displaced by extracellular Na^+^. Time constants for current changes in either high intracellular Na^+^ or K^+^ concentrations indicate that high intracellular K^+^ is able to reduce the effectiveness of the block of NaChBac by extracellular K^+^ by approximately 50% compared to high intracellular Na^+^ concentrations.

Many simulations indicate that sodium ions can “pass-by” one another in Na_v_ channels [12, 15, 16], not forming a tightly aligned “knock-on” arrangement as seen for K^+^ ions in potassium channels [6]. How multiple K^+^ ions traverse the selectivity filter of K^+^-channels under modest applied forces (0.5–1.0 kcal/mol/Å), however, is still controversial [32]. Our non-equilibrium pulling simulations using Na_v_Ab suggest that a “pass-by” configuration for potassium ions in the presence of external forces can block the selectivity filter in bacterial Na_v_ channels and prevent the permeation of K^+^ ions at small negative voltages in the inward direction, even though such a block state is unstable in the equilibrium free-energy landscape.

The parameter *λ* in the step-wise pulling protocol is used to incorporate driving forces due to applied voltages or salt concentration gradients in the simulations. When this parameter is used in harmonic potentials with large spring constants *k* >> *k*_0_ = 1 kcal/mol/Å^2^ [33], the step-wise pulling protocol with Jarzynski’s equality produces the same PMF as in umbrella sampling and WHAM methods [18, 19] which usually use large values of *k* (10 kcal/mol/Å^2^). To sample applied forces of the magnitude similar to biological membrane potentials (-100 mV to 100 mV), however, one must use *k* ~ *k*_0_ (~43 mV|e|/Å^2^). In step-wise pulling simulations another critical parameter is the relaxation time *τ*, i.e., the shorter relaxation time, the farther from equilibrium is the pulling sequence. We showed that when *τ* = 0.5 ns, three potassium ions can move more easily through the selectivity filter than sodium ions, even though they experienced an energy barrier (see Fig 2A). When *τ* = 3.0 ns, the movements of the potassium ions slow down and two potassium ions block the selectivity filter from S178 past E177. We estimated the work required to break the block and induce the inward currents to be 5–10 kcal/mol (equivalent to membrane potentials of -72 to -145 mV). These values coincide with the experimentally observed applied potentials for bacterial Na_v_ channels to induce inward currents of K^+^ ions in bionic conditions [11, 17].

**Fig 10:**
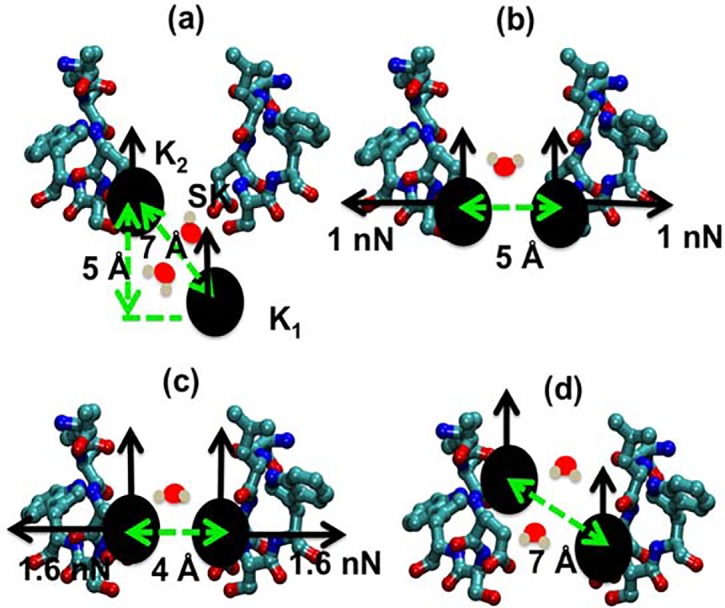
The configurations of two K^+^ ions in the process of inward permeation. (a) The ions in a local unperturbed free-energy minimum along the pathway (I) in Fig 8C. (b) The block state in an unstable unperturbed free-energy minimum denoted by Y in Fig 8C. (c) The two ions in another local unperturbed free-energy minimum denoted by X in Fig 8C. (d) The two ions about to enter the cavity. The dehydration of two K^+^ ions is illustrated by the reduction of shared water molecules between them from two (×2) to one (×2), where 2 is the symmetric factor. Note that only two of the four subunits of the channel are shown.

Fig 10 summarizes the movement of two potassium ions entering the block state and passing the selectivity filter in the inward direction. The Fig combines trajectories of the ions during the non-equilibrium simulations with the unperturbed equilibrium free-energy landscape. As shown in Fig 10A, a second K^+^ ion (K_1_) is found 5 Å below site SK along the z-axis of the channel, and four water molecules are shared between the two K^+^ ions. The distance between the two ions is approximately 5–7 Å, and this configuration corresponds to a global minimum in the two-dimensional PMF plot of two K^+^ ions in Fig 8C. We found that a modest external force F_ex_ in the pulling simulations (0.5–1 kcal/mol/Å = 0.035–0.07 nN) was sufficient to overcome the electrostatic repulsion of the two ions and to push the two K^+^ ions into the block configuration, with expulsion of the two water molecules (Fig 10B). Fig 10B is equivalent to the unstable local free-energy minimum denoted by (Y) in Fig 8C. Thermal fluctuations would easily push the ions from this configuration into any global minimum. The ions exert a force against opposite lateral walls of the channel that serves to wedge them together in a plane at the positions between E177 and L176 where width of the selectivity filter is approximately 4.6 Å wide, thus effectively blocking the inward K^+^ current. This only happens when there are the modest external forces such as experimental electric fields that have almost the same magnitude and act at the same time on the ions along the permeation pathway [11, 17]. When the applied forces gradually increase (*λ* from– 14 to -6 Å), the two K^+^ ions are pushed further inside the selectivity filter as shown in Fig 10C. In this configuration, although the repulsive force between the two ions becomes larger (≈1.6 nN), in fact the ions are in a global free-energy minimum (denoted by (X) in Fig 8C) due to the high negative charge density of carboxylate and carbonyl oxygen atoms (see Fig 4), thus converting them into the configuration shown in Fig 10D. When the ions enter the configurations shown in Fig 10C and 10D, the permeation process through the selectivity filter can be considered to be complete since in the absence of block, PMF results indicate that only 1–2 kcal/mol of energy is needed for the ions to pass though the selectivity filter from the extracellular surface of the channel to the central cavity.

The mechanism of block of Na_v_Ab by extracellular K^+^ described here can explain the functional asymmetry that was described by Finol-Urdaneta et al. for the NaChBac channel [17]. In that study, intracellular K^+^ was found to enhance the rate of replacement of bound extracellular K^+^ by extracellular Na^+^ in a wash-in wash-out procedure. Additionally, the instantaneous I-V plot of K current in the presence of symmetric high K^+^ concentrations was observed to have a reversal potential of zero mV, in contrast to the reversal potential of about -100 mV when intracellular K^+^ is replaced by Na^+^. If the high-affinity binding of K^+^ that was inferred from the observations by Finol-Urdaneta et al. occurs at the site SK in Fig 10A where two or three K^+^ ions block the channel as described here, then block would occur when the small external force associated with the K^+^ concentration gradient (high extracellular K^+^, low intracellular K^+^) is present, but would be absent when the concentration gradient for K^+^ is small or reversed. Our outward-pulling simulations further show that three potassium ions can move through the selectivity filter much more easily in the outward direction than the inward one. Apparently, the three amino acids LES can induce a block for the inward movement of potassium ions, but the three amino acids TLE favor the single-file configuration for the outward movement. Fig 6A suggests that the network of hydrogen bonds between glutamate and serine amino acids play a critical role in the block, while the carbonyl oxygen atoms of threonine, leucine and glutamate amino acids help forming a single file of potassium ions as seen in the sequence of KcsA (TTVGYG). The side chain of glutamate is also important for coordinating with serine in the block configuration. If mutating the glutamate with aspartic acid, it is likely that such a block would be absent, thus, the permeation of potassium ions in the inward direction is enhanced as observed in experiments[12]. Based on these results, it may be possible to block potassium ions in both directions by mutating TLESW to SEESW, thereby creating a very high selectivity of sodium over potassium ions.

The mechanism of extracellular K^+^ block of inward K^+^ current described above depends critically on the structure of the selectivity filter since the dimensions of the site SK (see Fig 10A) and the presence of glutamate carboxyl oxygen atoms will determine the affinity of the site for K^+^. The amino acid sequence TLESW that is found in the selectivity filter of Na_v_Ab is also conserved in Na_v_MS, NaChBac, and several other bacterial Na_v_ channels [9], and extracellular K^+^ has been found to block these channels in all experiments where it has been tested [11, 17]. The TLESW sequence is not conserved in the selectivity filter of most eukaryotic Na_v_ channels, however, and extracellular K^+^ is only modestly effective (< 20%) in blocking these channels [29]. Eukaryotic Na_v_ channels are blocked by extracellular H^+^, however, but it is unlikely that the mechanism of H^+^ block in eukaryotic Na_v_ channels is similar to the mechanism of K^+^ block in bacterial Na_v_ channels as described in this work.

In conclusion, this study describes a model for blocking the extracellular entrance of Na_v_Ab selectivity filter by potassium ions moving in the inward direction, but absent in the outward direction. The model explains a number of experimental observations. The work required to break the block or induce an inward current of potassium ions is estimated to be 5–10 kcal/mol, which is higher than the work to move the ions in the outward direction, in agreement with experiments of bacterial sodium channels. We found that that biasing external forces that are explicitly included in non-equilibrium simulations “lock” multiple potassium ions in an unstable local minimum that is observed in equilibrium unperturbed free-energy landscapes, but that this “lock” changes the dynamics of ion permeation predicted in the equilibrium simulations. Thus, the functional asymmetry that is observed in these channels can be attributed to block of the channels by extracellular K^+^ that occurs under the influence of external electrochemical forces and proper coordination of glutamate and serine amino acids.

## References

[1] Hille B. Ionic Channels of Excitable Membranes. 2 ed. Sunderland, MA: Sinauer Associates, Inc.; 1992 1992.

[2] Ashcroft FM. Ion Channels and Disease San Diego: Academic Press; 2000. 481 p.

[3] Doyle DA, Cabral JM, Pfuetzner RA, Kuo A, Gulbis JM, Cohen SL, et al The structure of the potassium channel: molecular basis of K conduction and selectivity. Science. 1998;280:69–77. 9525859 10.1126/science.280.5360.69

[4] Jiang Y, Lee A, Chen J, Ruta V, Cadene M, Chait BT, et al X-ray structure of a voltage-dependent K+ channel. Nature. 2003;423(6935):33–41. Epub 2003/05/02. 10.1038/nature01580 . 12721618

[5] Tao X, Lee A, Limapichat W, Dougherty DA, MacKinnon R. A gating charge transfer center in voltage sensors. Science. 2010;328(5974):67–73. Epub 2010/04/03. 10.1126/science.1185954 20360102 PMC2869078

[6] Zhou Y, Morais-Cabral JH, Kaufman A, MacKinnon R. Chemistry of ion coordination and hydration revealed by a K channel-Fab complex at 2.0 A resolution. Nature. 2001;414:43–8. 11689936 10.1038/35102009

[7] McCusker EC, Bagneris C, Naylor CE, Cole AR, D'Avanzo N, Nichols CG, et al Structure of a bacterial voltage-gated sodium channel pore reveals mechanisms of opening and closing. Nature communications. 2012;3:1102 Epub 2012/10/04. 10.1038/ncomms2077 23033078 PMC3493636

[8] Payandeh J, Gamal El-Din TM, Scheuer T, Zheng N, Catterall WA. Crystal structure of a voltage-gated sodium channel in two potentially inactivated states. Nature. 2012;486(7401):135–9. 10.1038/nature11077 22678296 PMC3552482

[9] Payandeh J, Scheuer T, Zheng N, Catterall WA. The crystal structure of a voltage-gated sodium channel. Nature. 2011;475(7356):353–8. 10.1038/nature10238 21743477 PMC3266868

[10] Zhang X, Ren W, DeCaen P, Yan C, Tao X, Tang L, et al Crystal structure of an orthologue of the NaChBac voltage-gated sodium channel. Nature. 2012;486(7401):130–4. 10.1038/nature11054 22678295 PMC3979295

[11] Ulmschneider MB, Bagneris C, McCusker EC, Decaen PG, Delling M, Clapham DE, et al Molecular dynamics of ion transport through the open conformation of a bacterial voltage-gated sodium channel. Proc Natl Acad Sci U S A. 2013;110(16):6364–9. 10.1073/pnas.1214667110 23542377 PMC3631666

[12] Furini S, Domene C. On conduction in a bacterial sodium channel. PLoS computational biology. 2012;8(4):e1002476 Epub 2012/04/13. 10.1371/journal.pcbi.1002476 22496637 PMC3320569

[13] Corry B. Na(+)/Ca(2+) selectivity in the bacterial voltage-gated sodium channel NavAb. PeerJ. 2013;1:e16 Epub 2013/05/03. 10.7717/peerj.16 23638350 PMC3629057

[14] Corry B, Thomas M. Mechanism of ion permeation and selectivity in a voltage gated sodium channel. J Am Chem Soc. 2012;134(3):1840–6. 10.1021/ja210020h . 22191670

[15] Chakrabarti N, Ing C, Payandeh J, Zheng N, Catterall WA, Pomes R. Catalysis of Na+ permeation in the bacterial sodium channel Na(V)Ab. Proc Natl Acad Sci U S A. 2013;110(28):11331–6. 10.1073/pnas.1309452110 23803856 PMC3710854

[16] Boiteux C, Vorobyov I, Allen TW. Ion conduction and conformational flexibility of a bacterial voltage-gated sodium channel. Proc Natl Acad Sci U S A. 2014;111(9):3454–9. 10.1073/pnas.1320907111 24550503 PMC3948317

[17] Finol-Urdaneta RK, Wang Y, Al-Sabi A, Zhao C, Noskov SY, French RJ. Sodium channel selectivity and conduction: prokaryotes have devised their own molecular strategy. J Gen Physiol. 2014;143(2):157–71. Epub 2014/01/15. 10.1085/jgp.201311037 . 24420772 PMC4001777

[18] Kumar S, Bouzida D, Swendsen RH, Kollman PA, Rosenberg JM. The weighted histogram analysis method for free-energy calculations on biomolecules. 1. The method. J Comp Chem. 1992;13:1011–21.

[19] Souaille M, Roux B. Extension to the weighted histogram analysis method: combining umbrella sampling with free energy calculations. Comput Phys Commun. 2001;135:40–57. 10.1016/S0010-4655(00)00215-0

[20] Humphrey W, Dalke A, Schulten K. VMD- Visual Molecular Dynamics. J Molec Graphics. 1996;14:33–8. 10.1016/0263-7855(96)00018-5 8744570

[21] Phillips JC, Braun R, Wang W, Gumbart J, Tajkhorshid E, Villa E, et al Scalable molecular dynamics with NAMD. Journal of computational chemistry. 2005;26(16):1781–802. 10.1002/jcc.20289 16222654 PMC2486339

[22] MacKerell AD, Bashford D, Bellott M, Dunbrack RL, Evanseck JD, Field MJ, et al All-atom empirical potential for molecular modeling and dynamics studies of proteins. The journal of physical chemistry B. 1998;102(18):3586–616. 10.1021/jp973084f . 24889800

[23] Mackerell AD Jr., Feig M, Brooks CL 3rd. Extending the treatment of backbone energetics in protein force fields: limitations of gas-phase quantum mechanics in reproducing protein conformational distributions in molecular dynamics simulations. Journal of computational chemistry. 2004;25(11):1400–15. 10.1002/jcc.20065 . 15185334

[24] Noskov SY, Berneche S, Roux B. Control of ion selectivity in potassium channels by electrostatic and dynamic properties of carbonyl ligands. Nature. 2004;431(7010):830–4. Epub 2004/10/16. 10.1038/nature02943 . 15483608

[25] Klauda JB, Venable RM, Freites JA, O'Connor JW, Tobias DJ, Mondragon-Ramirez C, et al Update of the CHARMM all-atom additive force field for lipids: validation on six lipid types. The journal of physical chemistry B. 2010;114(23):7830–43. 10.1021/jp101759q 20496934 PMC2922408

[26] Ngo V, Stefanovski D, Haas S, Farley RA. Non-equilibrium dynamics contribute to ion selectivity in the KcsA channel. PLoS One. 2014;9(1):e86079 Epub 2014/01/28. 10.1371/journal.pone.0086079 24465882 PMC3895005

[27] Ngo VA. Parallel-pulling protocol for free-energy evaluation. Physical review E, Statistical, nonlinear, and soft matter physics. 2012;85(3 Pt 2):036702. Epub 2012/05/17. . 22587204 10.1103/PhysRevE.85.036702

[28] Ngo VA, De Felice R, Haas S. Is the G-Quadruplex an Effective Nanoconductor for Ions? J Phys Chem B. 2014;118:864–72. 10.1021/jp408071h 24397412

[29] Hille B. Ionic selectivity, saturation, and block in sodium channels. A four barrier model. JgenPhysiol. 1975;66:535–60. 10.1085/jgp.66.5.535 PMC2226224 1194886

[30] Stock L, Delemotte L, Carnevale V, Treptow W, Klein ML. Conduction in a biological sodium selective channel. The journal of physical chemistry B. 2013;117(14):3782–9. 10.1021/jp401403b . 23452067

[31] Ke S, Timin EN, Stary-Weinzinger A. Different inward and outward conduction mechanisms in NaVMs suggested by molecular dynamics simulations. PLoS computational biology. 2014;10(7):e1003746 10.1371/journal.pcbi.1003746 25079564 PMC4117422

[32] Kopfer DA, Song C, Gruene T, Sheldrick GM, Zachariae U, de Groot BL. Ion permeation in K(+) channels occurs by direct Coulomb knock-on. Science. 2014;346(6207):352–5. 10.1126/science.1254840 . 25324389

[33] Park S, Schulten K. Calculating potentials of mean force from steered molecular dynamics simulations. The Journal of chemical physics. 2004;120(13):5946–61. 10.1063/1.1651473 . 15267476

